# Pregnancy-specific glycoprotein 9 (PSG9), a driver for colorectal cancer, enhances angiogenesis via activation of SMAD4

**DOI:** 10.18632/oncotarget.11146

**Published:** 2016-08-09

**Authors:** Lei Yang, Shusheng Hu, Jinjing Tan, Xiaojing Zhang, Wen Yuan, Qian Wang, Lingling Xu, Jian Liu, Zheng Liu, Yanjun Jia, Xiaoxi Huang

**Affiliations:** ^1^ Medical Research Center, Beijing Chaoyang Hospital, Capital Medical University, Beijing, P.R. China; ^2^ Clinical Laboratory Department, Tianjin Medical University Cancer Institute and Hospital, Tianjin, P.R. China; ^3^ Department of Cellular and Molecular Biology, Beijing Chest Hospital, Capital Medical University, Beijing, P.R. China; ^4^ Oncology Department, Beijing Chaoyang Hospital, Capital Medical University, Beijing, P.R. China

**Keywords:** colorectal cancer (CRC), angiogenesis, PSG9, SMAD4

## Abstract

PSG9 is a member of the pregnancy-specific glycoprotein (PSG) family and has been shown to contribute to the progression of colorectal cancer (CRC) and cancer-related angiogenesis. Here, we aim to investigate abnormal PSG9 levels in patients with CRC and to emphasize the role of PSG9 in driving tumorigenesis. Serum from 140 patients with CRC and 125 healthy controls as well as 74 paired tumors and adjacent normal tissue were used to determine PSG9 levels. We discovered that PSG9 was significantly increased in serum (*P*<0.001) and in tumor tissues (*P*<0.001) from patients with CRC. Interestingly, the increased PSG9 levels correlated with poor survival (*P*=0.009) and microvessel density (MVD) (*P*=0.034). The overexpression of PSG9 strongly promoted the proliferation and migration of HCT-116 and HT-29 cells. However, PSG9 depletion inhibited the proliferation of SW-480 cells. Using a human umbilical vein endothelial cell tube-forming assay, we found that PSG9 promoted angiogenesis. The overexpression of PSG9 also increased the growth of tumor xenografts in nude mice. Co-immunoprecipitation experiments revealed that PSG9 was bound to SMAD4. The PSG9/SMAD4 complex recruited cytoplasmic SMAD2/3 to form a complex, which enhanced SMAD4 nuclear retention. The PSG9 and SMAD4 complex activated the expression of multiple angiogenesis-related genes (included *IGFBP-3, PDGF-AA, GM-CSF*, and *VEGFA*). Together, our findings illustrate the innovative mechanism by which PSG9 drives the progression of CRC and tumor angiogenesis. This occurs via nuclear translocation of PSG9/SMAD4, which activates angiogenic cytokines. Therefore, our study may provide evidence for novel treatment strategies by targeting PSG9 in antiangiogenic cancer therapy.

## INTRODUCTION

PSG9 is a member of the pregnancy-specific glycoprotein (PSG) family. PSGs are encoded by ten *PSG* genes (*PSG1-PSG9, PSG11*) and exhibit sequence similarity to the carcinoembryonic antigen (CEA) cell adhesion molecule (CEACAM) family [1, 2]. CEA has been widely used as a cancer marker for the clinical management of colorectal cancer (CRC), and elevated levels of CEA in the plasma/serum indicate metastasis and poor prognosis [3]. However, few studies have revealed the involvement of PSGs in tumor-related processes.

Previous studies have recognized PSGs as secreted proteins that are the most abundant trophoblastic proteins in maternal blood during human pregnancy [4]. Human PSGs are heavily N-glycosylated proteins that consist of four immunoglobulin (Ig)-like domains, an amino-terminal Ig variable-like (N) domain and three Ig constant-like (C) domains [5]. PSG9 has a different protein structure from other PSG family members, as it lacks an RGD (Arg-Gly-Asp)-motif [6]. Several studies have indicated that PSGs have a proangiogenic function because their expression induces endothelial tube formation [7]. Recently, PSG1 was demonstrated to activate the anti-inflammatory cytokines transforming growth factor (TGF)-β1 and TGF-β2 [4]. However, other types of PSGs still failed to act as biological activators. The abnormally high levels of PSG9 detected in cases of familial adenomatous polyposis (FAP) and in adenomas may promote colorectal carcinogenesis [8]. However, the function and pathobiological importance of PSG9 remain to be determined.

CRC ranks as the fifth most common cancer in males and as the fourth most common cancer in females in China [9]. In comparison, CRC is the fourth most common cancer diagnosed in the United States. CRC continues to demonstrate a significant upward trend and is a leading cause of cancer-related deaths [9, 10]. The 5-year survival rates have remained at approximately 10% for patients with stage IV CRC and distant metastasis [11]. Almost 50% of patients with CRC will develop metastases, and 25% of cases already have metastatic disease at diagnosis [12]. The tumor vasculature is pivotal to tumor growth and metastases because tumor cells require nutrients and oxygen from nearby capillaries to function and survive [13]. Furthermore, blood vessels provide pivotal channels that permit metastatic cancer cells to reach distal organs [14]. Unlike physiological angiogenesis, which is tightly controlled, tumor vessels are disorganized, tortuous, immature, fragile and leaky [15, 16].

The TGF-β signaling pathway can induce a proangiogenic environment, as it stimulates tumor angiogenesis [17]. TGF-β1 plays an important role in the maintenance of epithelial homeostasis, including the processes of proliferation, differentiation, migration and apoptosis [18]. SMAD proteins are recognized as central mediators of TGF-β signaling pathways, which promote angiogenesis through the activation of transcription of platelet-derived growth factor (PDGF)-B [19]. SMAD4 binds to SMAD2/3 and forms a heteromeric complex, which then translocates to the nucleus, where it binds to intended gene promoters [20]. SMAD4 expression in cancer cells was confirmed to positively correlate with enhanced microvessel density (MVD) [21].

In the present study, we first revealed the overexpression of PSG9 in patients with CRC. Then, we further investigated potential crosstalk between PSG9 and TGF-β/SMAD4 signaling, which is an intensively studied oncogenic pathway. We also explored whether PSG9 is a key component in the regulation of TGF-β signaling-induced tumor angiogenesis via enhancement of nuclear retention of SMAD4, and finally, we determined whether the expression of proangiogenic molecules is affected by the PSG9/SMAD4 interaction.

## RESULTS

### High PSG9 levels in serum from CRC patients

**Figure 1:**
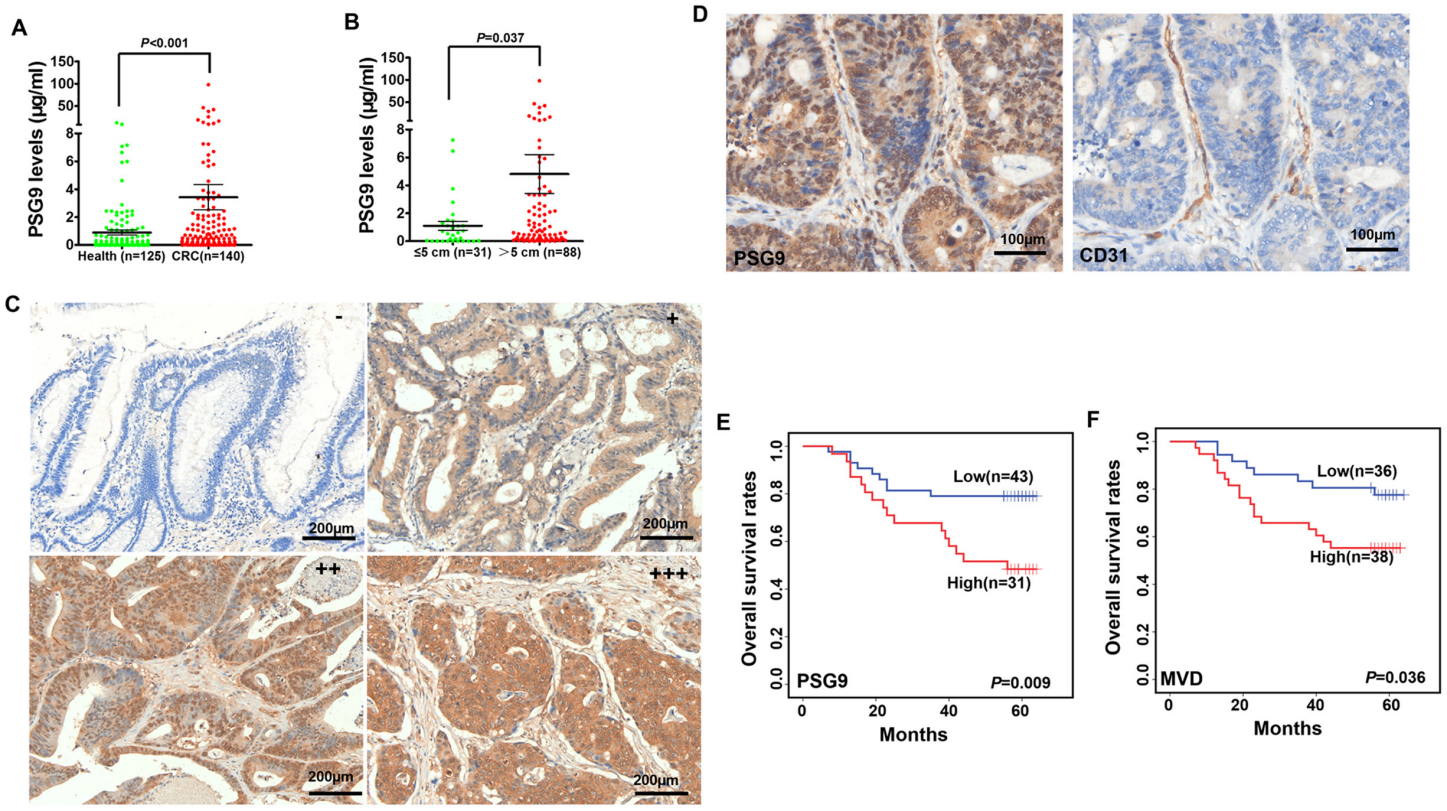
High levels of PSG9 are associated with micro-vessel density (MVD) and poor outcomes in patients with colorectal cancer (CRC) **A.** PSG9 levels in serum from healthy controls and patients with CRC. **B.** PSG9 levels in CRC patients with a tumor diameter ≤ 5 cm and > 5 cm. **C.** The levels of PSG9 range from negative (−) (score of 0~2 score), weak (+) (score of 3~4), moderate (++) (score of 4) to strong (+++) (score ≥ 5) in CRC tumors. **D.** CD31 and PSG9 immunohistochemical staining in a CRC tissue section illustrates the location of PSG9 and the MVD. Kaplan-Meier curves show the differences between the high and low expression of PSG9 (E) and MVD (F).

We searched the Oncomine microarray database to analyze the expression of PSGs in CRC patients. Four (PSG1, PSG3, PSG6, and PSG9) of 11 PSG members showed differential mRNA expression levels in tissues from CRC patients (Supplementary Figure S1). The fold change in PSG9 expression was the highest out of all PSGs (Supplementary Figure S1). We further tested the levels of circulating PSG9 proteins in serum samples and found abnormally high PSG9 levels in the serum of CRC patients compared with serum from healthy controls (Figure 1A, Supplementary Table S1). Moreover, CRC patients with tumors > 5 cm showed higher serum PSG9 levels than those with tumors ≤ 5 cm, and it was found that PSG9 had the potential to promote tumor cell proliferation (Figure 1B, Supplementary Table S1).

### High PSG9 levels are positively associated with MVD and predict a shorter OS time

To analyze PSG9 expression in CRC tissues, a tissue microarray was stained with a PSG9 antibody. Finally, 74 subjects were enrolled for subsequent analysis. Negative, weak and moderate PSG9 staining was regarded as low expression, while strong PSG9 staining was regarded as high expression (Figure 1C, Supplementary Figure S2A). In cancer tissues, 31 (41.89%) cases displayed high PSG9 expression. Three (4.05%) cases showed strong PSG9 staining in adjacent normal tissues (Table 1). This indicated that CRC tissues had significantly higher PSG9 levels than adjacent normal tissues (Table 1). The analysis of the relationship between PSG9 and the pathological characteristics of the tumors is summarized in Supplementary Table S2.

**Table 1:** PSG9 levels in cancer and adjacent normal tissues from 74 CRC patients

	PSG9 staining				*χ^2^*	*P* value
	-	+	++	+++		
T (n=74)	2 (2.70%)	15 (20.27%)	26 (35.14%)	31 (41.89%)	60.24	<0.001
N (n=74)	38 (51.35%)	26 (35.14%)	7 (9.46%)	3 (4.05%)		

The MVD in 74 CRC tissues was analyzed by staining for CD31. The microvessels stained by the CD31 antibody were distributed within the mesenchymal tissues surrounding the carcinoma nests (Figure 1D, Supplementary Figure S2B). Therefore, we concluded that PSG9 might be secreted by cancer cells into the mesenchyme, where it enhances angiogenesis. Next, we compared the difference in MVD in patients with CRC whose tumors demonstrated high and low PSG9 expression. In all, 64.52% (20/31) of CRC patients whose tumors had high PSG9 expression showed higher MVD than those whose tumors had low PSG9 expression; of these, 39.53% (17/43) of cases displayed low MVD (Supplementary Figure S2C, Supplementary Table S3). We thus deduced that PSG9 might promote angiogenesis in CRC tissues.

Furthermore, 9 (20.93%) patients out of 43 with low PSG9 expression had died. However, 16 (51.61%) out of 31 subjects with high PSG9 expression had died. A Kaplan-Meier analysis revealed that the overall survival rates in the high PSG9 expression group were significantly shorter than those in the low expression group (Figure 1E). Kaplan-Meier analysis also demonstrated that CRC patients whose tumors featured low MVD had a longer overall survival time than those whose tumors featured high MVD (Figure 1F). A Cox univariate regression analysis also revealed a shorter cumulative survival in patients with high levels of PSG9 or MVD (Supplementary Table S4).

### PSG9 promotes the proliferation and migration of CRC cells

**Figure 2:**
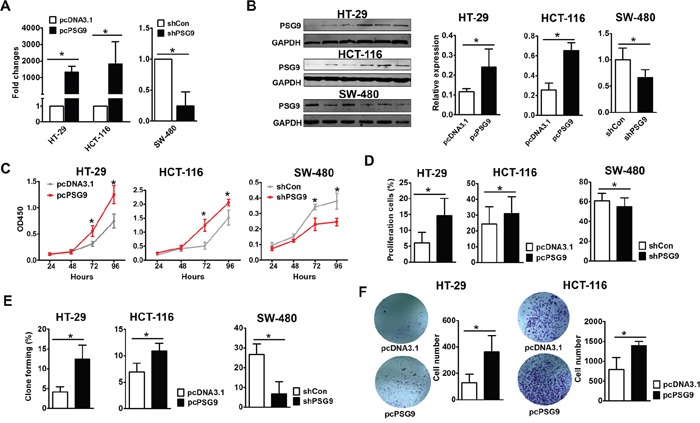
PSG9 promotes the proliferation and migration of CRC cells **A.** Quantitative real-time PCR detected the expression of PSG9 in HT-29/HCT-116-pcDNA3.1/pcDNA-3.1-PSG9 (pcPSG9) and SW-480-shControl (shCon) and shPSG9 cells. The data represent the mean±S.D. values of three replicates. *P<0.05. **B.** Western blot determined the expression of PSG9 in HT-29/HCT-116-pcDNA3.1/pcPSG9 cells and SW-480-shCon/shPSG9 cells. Cells transfected with the pcPSG9 plasmid displayed significantly higher PSG9 expression compared with cells that were transfected with the pcDNA3.1 plasmid. Conversely, PSG9 shRNA decreased the expression of PSG9 in SW-480 cells. **C.** A CCK-8 assay was used to analyze the proliferation of HT-29 and HCT-116 cells stably transfected with pcDNA3.1 and pcPSG9 and in SW-480 cells stably transfected with shCon and shPSG9. **D.** EdU incorporation assays compared the percentage of proliferating cells in HT-29/HCT-116-pcPSG9 and HT-29/HCT-116-pcDNA3.1 cells as well as in SW480 cells stably transfected with shPSG9 and shCon. **E.** Colony formation assays revealed the colony formation rates of HT-29/HCT-116-pcDNA3.1 and HT-29/HCT-116-pcPSG9 cells as well as SW-480-shCon and SW-480-shPSG9 cells. **F.** Transwell migration assays determined the migration abilities of HT-29/HCT-116-pcDNA3.1 and HT-29/HCT-116-pcPSG9 cells. The data represent the mean±S.D. values of three replicates. *P<0.05.

We proposed that PSG9 might be one of the drivers that promotes the progression of CRC during carcinogenesis. We thus determined whether the change in PSG9 expression effects the proliferation of CRC cells. We selected HT-29 and HCT-116 cells to upregulate PSG9, which have relatively low PSG9 levels, and SW-480 cells to downregulate PSG9, which have high PSG9 levels, respectively (Supplementary Figure S3A, Figure 2A, 2B). Initially, CCK8 assays demonstrated that stable transfection of HT-29 and HCT-116 cells with PSG9 resulted in higher proliferation rates than the stable transfection of these cells with empty vectors (Figure 2C). However, PSG9 downregulation in SW-480 cells led to a lower proliferation rate compared with SW-480 cells that were stably transfected with control shRNA (Figure 2C). EdU incorporation assays were then performed to analyze the proportion of proliferating cells (Supplementary Figure S3B). As shown in Figure 2D, the overexpression of PSG9 significantly increased the proportion of proliferating HT-29 and HCT-116 cells. Furthermore, we also found that transfection with PSG9 shRNA significantly decreased the number of proliferating SW-480 cells compared with the stable transfection of these cells with control shRNA (Figure 2D). The number of clones that formed in soft agar after stable transfection of HT-29 and HCT-116 cells with PSG9 and after stable transfection of SW-480 cells with PSG9 shRNA was calculated (Supplementary Figure S3C). As expected, the upregulation of PSG9 significantly enhanced the colony formation rates (Figure 2E). On the contrary, the downregulation of PSG9 decreased the colony formation rate of SW-480 cells (Figure 2E). Next, by Transwell assay, we examined whether PSG9 plays a role in the migration of CRC cells. Overexpression of PSG9 significantly strengthened the migration of HT-29 and HCT-116 cells compared with the controls (Figure 2F).

### PSG9 promotes tumor proliferation in an animal tumor xenograft model via enhancement of angiogenesis

**Figure 3:**
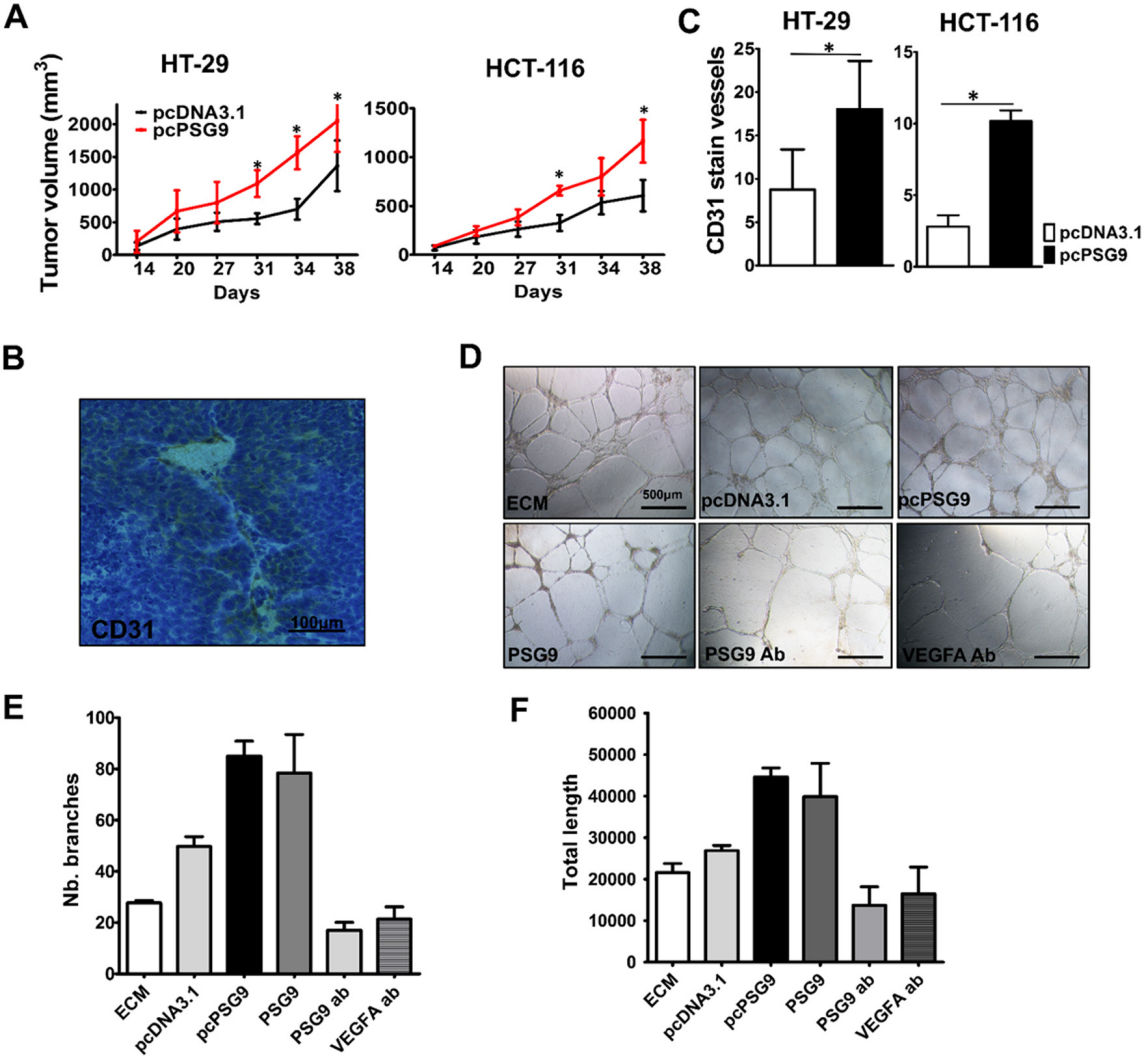
PSG9 promotes tumor proliferation *in vivo* and enhances angiogenesis *in vivo* and *in vitro* **A.** Tumor xenografts from nude mice that were injected with HT-29-pcPSG9 cells (n=5) exhibited significantly higher growth rates compared with tumors from mice that were injected with HT-29-pcDNA3.1 cells (n=4). Tumors from mice that were injected with HCT-116-pcPSG9 cells (n=5) also exhibited increased growth rates compared with tumors from mice injected with HCT-116-pcDNA3.1 cells (n=5). *P<0.05. **B.** Representative immunohistochemical staining for CD31 imaged from HT-29-pcPSG9 cells that formed tumor xenografts. **C.** IHC staining for CD31 in the blood vessels of tumor xenografts derived from HT-29/HCT-116-pcPSG9 cells was significantly higher than staining in tumors derived from HT-29/HCT-116-pcDNA3.1 cells. *P<0.05. **D.** Endothelial cell medium (ECM) and conditioned medium (CM) collected from HCT-116-pcDNA3.1 and HCT-116-pcPSG9 cells, along with 5 μg/ml purified human recombinant PSG9 proteins, PSG9 antibody (ab), and VEGFA ab were used to induce the formation of vessel-like tubes in HUVECs. **E.** pcPSG9 CM and PSG9 proteins increased the number (Nb.) of HUVECs that formed branches compared with treatment with HCT-116-pcDNA3.1 CM, ECM, PSG9 ab, and VEGFA ab. **F.** pcPSG9 CM and PSG9 proteins led to a greater total length of vessel-like tubes that formed in HUVECs. The data (Nb. branches and length) represent the mean±S.D. values of three replicates.

Next, we asked whether PSG9 overexpression promotes the formation of tumor xenografts. We injected HT-29 and HCT-116 cells that were stably transfected with pcDNA3.1 and pcDNA3.1-PSG9 into BALB/c nude mice (Supplementary Figure S4A). As expected, the upregulation PSG9 either in HT-29 or HCT-116 cells resulted in significantly higher proliferation rates for tumor xenografts in mice (Figure 3A). We speculated that PSG9 enhanced angiogenesis in this case and thus provided a precondition for rapid tumor growth. The xenografted tumor tissues were subjected to immunohistochemistry (IHC) for CD31 to show the microvasculature (Figure 3B). We detected more vessels in tumor xenografts from mice injected with stable PSG9-transfected HT-29 or HCT-116 cells (Figure 3C). These findings indicated that PSG9 induces tumor cells to construct more vessel channels.

Furthermore, we detected PSG9 and VEGFA expression in HUVECs by immunofluorescence (IF). It was found that PSG9 and VEGFA were distributed in the cell membrane, cytoplasm and nucleus of HUVEC cells (Supplementary Figure S4B). Through a HUVEC Matrigel tube-forming assay, we also validated that the upregulation of PSG9 enhances sprouting angiogenesis *in vitro*. HUVECs were cultured with endothelial cell media (ECM) or conditioned media (CM) from HCT-116-pcDNA3.1 cell culture and HCT-116-pcPSG9 cell culture for 24 hours. Besides, we produced recombinant human PSG9 proteins (Supplementary Figure S4C, S4D). For this assay, HUVECs were treated with 5 μg/ml PSG9, anti-PSG9 antibody, and anti-VEGFA antibody for 24 hours (Figure 3D). As a result, the use of PSG9 CM and protein media increased the number of tube branches of HUVECs. Conversely, the number of branches was reduced when HUVECs were treated with antibodies against PSG9 and VEGFA (Figure 3E). Similarly, the culture of HUVECs with PSG9 CM and protein media prolonged tube length, whereas the culture of HUVECs with media containing PSG9 and VEGFA antibodies decreased tube length (Figure 3F). Thus, we can conclude that PSG9 may function as a proangiogenic factor.

### PSG9 stimulates the TGF-β pathway by directly binding to SMAD4

**Figure 4:**
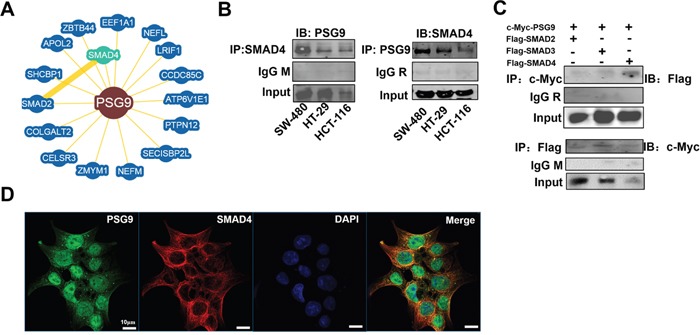
PSG9 binds directly to SMAD4 **A.** BioGRID^3.4^ identified 16 proteins bound to PSG9. SMAD4 is the closest molecule to PSG9 in the protein-protein interaction network. **B.** A co-immunoprecipitation (Co-IP) assay was performed to determine the interaction between PSG9 and SMAD4. Left, anti-SMAD4 antibody was used for PSG9 immunoprecipitation (IP), and a mouse IgG (IgG M) antibody was used as the negative control. Immunoblotting (IB) with a PSG9 antibody was used to determine the PSG9 levels in SW-480, HT-29, and HCT-116 cells. Right, anti-PSG9 antibody was used for SMAD4 immunoprecipitation, and a rabbit IgG (IgG R) antibody was used as the negative control. **C.** Exogenous expression of PSG9 and SMAD2/3/4 in HCT-116 cells. Vectors of c-myc-pcDNA3.1 PSG9 combined with Flag-SMAD2, Flag-SMAD3 or Flag-SMAD4 were transfected into HCT-116 cells for 24 hours. Immunoblotting with an anti-Flag antibody detected higher levels of Flag-SMAD4 than Flag-SMAD2/3 when an anti-c-myc antibody was subjected to Flag-SMAD2/3/4 immunoprecipitation (Top). An anti-Flag antibody subjected to c-myc-PSG9 and anti-c-myc antibody was used to immunoblot for PSG9 (Bottom). **D.** IF shows the location of the PSG9 and SMAD4 proteins in HCT-116 cells. DAPI was used to stain the nucleus.

To understand the involvement of PSG9 in signaling pathways, we determined the proteins with which PSG9 interacts. We screened 16 proteins through a bioinformatics analysis using BioGRID^3.4^ (Figure 4A). Among of them, SMAD4, a key signaling molecule in TGF-β signaling, was determined to be one of the proteins that most likely interacts with PSG9. We considered that PSG9 might bind directly to SMAD4 to activate the TGF-β signaling pathway. Next, co-immunoprecipitation (Co-IP) assays were used to verify the physiological interaction between PSG9 and SMAD4. The endogenous association of PSG9 with SMAD4 was detected in SW-480, HT-29, and HCT-116 cells (Figure 4B). As SMAD2/3/4 forms a trimer, which activates TGF-β signaling, we tested whether PSG9 affects this complex. The vectors that express full-length, flag-tagged SMAD2, SMAD3, or SMAD4 were each transfected into HCT-116 cells. At the same time, cells were also transfected with a c-myc-pcDNA3.1-PSG9 vector. Next, Co-IP assays demonstrated that c-myc-pcDNA3.1-PSG9 showed a higher affinity to Flag-SMAD4 than to Flag-SMAD2 or Flag-SMAD3 (Figure 4C). The Flag antibody was used to precipitate exogenous PSG9, which confirmed the interaction of PSG9 with SMAD2/3/4 (Figure 4C). The above data indicated that PSG9 first binds to SMAD4, and then, PSG9/SMAD4 recruits SMAD2 and SMAD3 to form a complex.

An IF assay was used to show the location of PSG9 and SMAD4 in HCT-116 cells. The accumulated PSG9 in the cytoplasm might be transported though vesicles, as the majority of SMAD4 protein was found in the cytoplasm (Figure 4D). Thus, we propose that PSG9 binds to SMAD4 in the cytoplasm, then recruits SMAD2/3 to form a complex, which activates TGF-β signaling and translocates to the nucleus.

### PSG9 increases the nuclear retention of SMAD4 through maintenance of the SMAD2/3/4 complex

**Figure 5:**
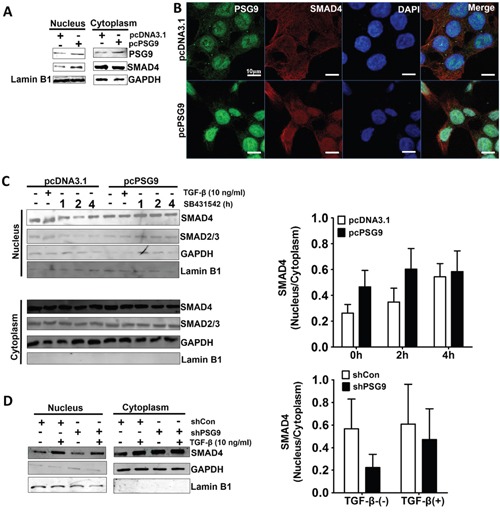
PSG9 increases the nuclear retention of SMAD4 **A.** PSG9 increased the nuclear retention of SMAD4 in stable transfected pcPSG9 HT-29 cells compared with stable transfected pcDNA3.1 cells. **B.** SMAD4 nuclear expression was determined by IF in HCT-116-pcDNA3.1 and HCT-116-pcPSG9 cells after treatment with 10 ng/ml TGF-β for 4 hours. **C.** PSG9 increased the nuclear retention of SMAD4 in HCT-116 cells. HCT-116-pcDNA3.1 and HCT-116-pcPSG9 cells were treated with 10 ng/ml TGF-β for 4 hours; cells were washed 3 times to remove TGF-β and were then treated with SB431542 for up 4 hours. The cells were harvested at the indicated times, and both the nuclear and cytoplasmic fractions were collected. SMAD4 nuclear and cytoplasmic ratios were analyzed after treatment with SB431542. Data are expressed as the mean±S.D. values from three replicates. **D.** Depletion of PSG9 reduced the levels of nuclear SMAD4 compared with shCon in SW-480 cells. SW-480 cells were treated with 10 ng/ml TGF-β for 4 hours. Data are expressed as the mean±S.D. values from 2 experiments.

Nuclear localization of SMAD4 and its direct binding to intended gene promoters are necessary for TGF-β signaling-associated angiogenesis. To understand the molecular mechanism of PSG9 activation of SMAD4, we first compared the levels of SMAD4 in cells with PSG9 upregulation and control cells, but we did not detect any changes (data not shown). Following this, we compared the levels of SMAD4 in HT-29 cells stably transected with PSG9 and HT-29 cells stably transfected with control vector. We found that the nuclear localization of SMAD4 was increased, but no changes were detected in the levels of SMAD4 in the cytoplasm (Figure 5A). IF staining for PSG9 and SMAD4 also revealed that HCT-116-pcPSG9 had increased nuclear localization of SMAD4 under TGF-β treatment (Figure 5B).

The location of SMAD4 in the cytoplasmic and nuclear fractions of TGF-β- treated HCT-116-pcDNA3.1 and HCT-116-pcPSG9 cells was examined in the presence of the TGF-β-receptor I (TβRI) inhibitor SB431542, which blocked SMAD3 and SMAD4 nuclear translocation. By Western blot of HCT-116-pcDNA3.1 and HCT-116-pcPSG9, we found that the PSG9 level in the nucleus was higher than that in the cytoplasm (Figure 5C). TGF-β treatment also increased the nuclear localization of SMAD4. In contrast, SB431542 treatment decreased the level of nuclear SMAD4 in HCT-116-pcDNA3.1 cells, while the upregulation of PSG9 enhanced the nuclear localization of SMAD4 in HCT-116-pcPSG9 cells. In other words, the upregulation of PSG9 antagonized the action of SB431542 on the reduction of SMAD4 nuclear translocation (Figure 5C). The same findings were also shown for SMAD2/3 (Figure 5C). Conversely, the knockdown PSG9 decreased the nuclear retention of SMAD4 in SW-480 cells (Figure 5D).

### PSG9/SMAD4 induces the expression of angiogenic factors

**Figure 6:**
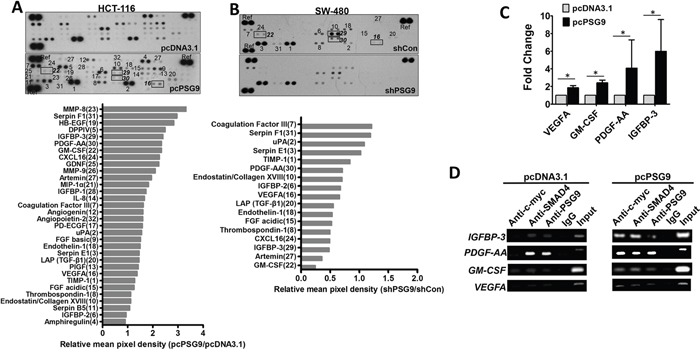
PSG9/SMAD4 induces the expression of angiogenesis-related genes **A.** An angiogenesis membrane-based antibody array was used to determine human angiogenesis-related proteins in HCT-116-pcDNA3.1 and HCT-116-pcPSG9 cell lysates. **B.** An antibody array was used to test the levels of angiogenesis-related proteins in SW-480-shControl (Con) and SW-480-shPSG9 cell lysates. Bottom, semi-quantitative data from dot density relative reference (Ref). **C.** Quantitative PCR determined the mRNA levels of *VEGFA, PDGF-AA, GM-CSF*, and *IGFBP-3*. The data represent the mean±S.D. values of at least four replicates. *P<0.05. **D.** Chromatin immunoprecipitation (ChIP) revealed that the PSG9/SMAD4 complex was bound to the promoters of *IGFBP-3, PDGF-AA, GM-CSF*, and *VEGFA* in HCT-116-pcDNA3.1 and HCT-116-pcPSG9 cells.

It is well known that SMAD4/TGF-β signaling activates many angiogenesis- related genes by binding to their promoter regions. Next, an angiogenesis membrane-based antibody array was used to screen differential proteins whose expression is affected by PSG9. Antibody arrays allowed us to detect 58.18% (32/55) of angiogenic proteins in HCT-116 cells (Figure 6A). The protein mean pixel density relative to the reference was calculated, which revealed that the upregulation of PSG9 increased the levels of MMP-8, Serpin F1, HB-EGF, DDPIV, IGFBP-3, PDGF-AA, GM-CSF, CXCL16, GNDF, and MMP-9 with changes greater than 2-fold in HCT-116-pcPSG9 cells relative to HCT-116-pcDNA3.1 cells (Figure 6A). Furthermore, the downregulation of PSG9 decreased the levels of GM-CSF, Artemin, IGFBP-3, CXCL16, Thrombospondin-1, FGF acidic, Endothelin-1, TGF-β1, VEGFA, and PDGF-AA less than 0.8-fold in SW-480-shPSG9 cells compared with SW-480-shCon cells (Figure 6B).

Next, we selected four proteins (IGFBP-3, PDGF-AA, GM-CSF, and VEGFA), that might represent the genes that are most likely activated by PSG9 and further determined their mRNA levels in HCT-116-pcDNA3.1 and HCT-116-pcPSG9 cells. Stable transfection of cells with PSG9 increased the mRNA levels of the *IGFBP-3, PDGF-AA, GM-CSF*, and *VEGFA* genes (Figure 6C). A chromatin immunoprecipitation (ChIP) assay was used to determine whether promoters of *IGFBP-3, PDGF-AA, GM-CSF*, and *VEGFA* were bound by endogenous SMAD4/PSG9 proteins in HCT-116-pcDNA3.1 and HCT-116-pcPSG9 cells. Antibodies specific to SMAD4, c-myc, and PSG9 were applied to immunoprecipitate the promoters of *IGFBP-3, PDGF-AA, GM-CSF*, or *VEGFA* in HCT-116-pcDNA3.1 and HCT-116-pcPSG9 cells (Figure 6D). We found that stable transfection of cells with PSG9 strengthened the affinity of the SMAD4/PSG9 complex to the promoters of *IGFBP-3, PDGF-AA, GM-CSF*, and *VEGFA* (Figure 6D). Collectively, these results indicated that PSG9/SMAD4 binds to the promoters of multiple angiogenic genes to activate their expression.

## DISCUSSION

We report here for the first time that through an enhancement of angiogenesis, PSG9 is a potential driver of CRC. High PSG9 levels were found to be related to increased tumor growth. Importantly, elevated PSG9 levels in CRC patients were significantly associated with poor outcomes after surgery.

Angiogenesis induction is pivotal to tumor growth and metastases [14]. Our findings indicate that PSG9 is one mediator of angiogenesis induction. Disturbances in angiogenesis provide the most important precondition for tumor cell migration and results in an unfavorable outcome. Tumor-stroma interactions are indispensable participants in the metastatic process [22]. Neo-vessel sprouting in vascular tubes was observed following continuous growth of endothelial cells in the stroma. Upregulated PSG9 in tumor cells might be secreted into the stroma through vesicular transport, where it affects endothelial cell growth in a paracrine manner.

Previously, a large amount of data supported an early tumor-suppressive role for TGF-β. However, it was also shown that TGF-β signaling could promote tumor progression and metastasis [23]. However, it is still not known how TGF-β signaling switches from tumor suppressor to tumor promoter. SMAD4 is one of the important components in the induction of TGF-β signaling and has been reported to be a tumor suppressor [24]. Approximately 20-30% of patients with CRC had SMAD4 mutations [20, 25]. These mutations might prevent the normal function of SMAD4, and therefore, tumor growth is not suppressed. Recently, Hernanda *et al.* demonstrated that SMAD4 exerted a tumor-promoting role in hepatocellular carcinoma (HCC) [20]. Our results reveal that PSG9 directly binds to SMAD4 to form a complex that enhances the nuclear retention of SMAD4. Whether mutation is the major reason for this drastic nuclear retention of PSG9/SMAD4 requires further exploration in future studies. TGF-β binds to its type 2 transmembrane receptor (TGFBR2), which recruits SMAD2 and SMAD3. This event allows for complexing with SMAD4 and subsequent nuclear translocation [26]. Here, we demonstrated that PSG9 binds directly to SMAD4, but that PSG9 does not alter the expression of SMAD4. The PSG9/SMAD4 complex promotes complexing with SMAD2/3, and the generation of this complex accelerates nuclear translocation. Considering that PSG9 binds to SMAD4, which promotes the transcription of proangiogenic factors, we reasoned that nuclear retention of PSG9/SMAD4 is an important mechanism in the promotion of angiogenesis.

TGF-β is sufficient to upregulate the expression of multiple cytokines, which in turn attracts neighboring endothelial cells and promotes angiogenesis [26, 27]. Our results demonstrate that nuclear PSG9/SMAD4 binds to promoters and activates the transcription of multiple angiogenic cytokines, including IGFBP-3, PDGF-AA, GM-CSF, and VEGFA. IGFBP-3 is a major IGF-binding protein in humans, and it enhances angiogenic activity in cancer through both IGF-dependent and IGF-independent mechanisms [28]. PDGF-AA is one of the major angiogenic mediators that has been shown to influence endothelial cell function through SMAD4 [29]. Moreover, the reason for the failure of anti-VEGF treatment is mostly likely because of the activation of PDGF-associated pathways [30]. Therefore, the poor prognosis related to PSG9 in our study might be partially explained by the PSG9-induced activation of PDGF pathways, which promote angiogenesis. Reports that PDGF-AA promotes cancer cell proliferation are consistent with our findings of PSG9 function on cell proliferation [31]. GM-CSF is a pro-inflammatory cytokine that is highly expressed in CRC and stimulates the growth and migration of human colorectal cancer cells. GM-CSF has also been found to be overexpressed in CRC [32] and is involved in the activation of several intracellular signaling pathways, including JAK/STAT, RAS/ERK, and PI3K/AKT [33]. Tumor-derived GM-CSF appears to drive the epithelial secretion of VEGF via an autocrine or paracrine mechanism [34]. Recently, PSG9 was proved to stimulate increase in FoxP3+ regulatory T cells through the TGF-β1 pathway [35]. It was not only consistence with our finding that PSG9 activated the TGF-β pathway, but also proved that PSG9 limited anti-tumor immunity and polarized microenvironment to a more pro-angiogenic microenvironment. The activation of these proangiogenic factors and the subsequent promotion of angiogenesis by PSG9/SMAD4 indicate a potential treatment approach (inhibition of PSG9) for patients with advanced CRC. The crosstalk between PSG9/SMAD4 and angiogenic factors needs to be further analyzed in future studies

In summary, our findings indicate that the interaction of PSG9 with the transcription factor SMAD4 promotes its functions and is therefore critical for TGF-β-activated expression of angiogenesis-associated genes, cell growth, and cancer metastasis. Our findings that the overexpression of PSG9 promotes cancer cell growth and vasculogenesis provide insights into mechanisms and strategies for therapeutic intervention in advanced CRC.

## MATERIALS AND METHODS

### Serum samples

Serum samples were collected from 140 patients with CRC prior to surgery and from 125 healthy volunteers at the Tianjin Medical University Cancer Hospital from May 2014 to December 2014. All donors provided informed consent, and the study was conducted under the approval of the Institutional Ethics Committee. The concentration of PSG9 proteins in the serum samples was measured by an ELISA kit (CUSABIO, Wuhan, China) according to the manufacturer's instructions.

### Immunohistochemical staining analysis

The HCol-Ade180Sur-07 tumor tissue microarray (TMA) (Shanghai OUTDO Biotech Co., Shanghai, China) consisted of 90 paired colorectal adenocarcinoma tissues and matched normal mucosa; this TMA included 74 completed cases and 16 censored cases. Patients underwent surgery from January 2009 to October 2009, and the follow-up information was available from February 2009 to May 2014. The TMA slide was de-paraffinized and rehydrated. The slide was pre-treated with EDTA antigen retrieval solution (pH=8.0) and rinsed in water. The sections were then blocked in 2% goat serum and were incubated with the primary antibody overnight at 4°C. The PSG9 staining intensity and percentage of stained cells were analyzed as described previously [36]. The intensity of the chromogen was graded as 0 (no color), 1 (light yellow), 2 (light brown), or 3 (brown) and the number of positive cells was graded as 0 (<5%), 1 (5-25%), 2 (25-50%), 3 (51-75%), or 4 (>75%). The two values were summed, and specimens were assigned to one of 4 levels as follows: score of 0~2 score (−), score of 3~4 (+), score of 4 (++), or a score of ≥ 5 (+++).

The hotspot method was utilized to assess the microvessel density (MVD) on the CD31-stained tumor tissue sections as previously descried [37, 38]. MVD was measured in three to five fields with a higher density of CD31-positive cells and cell clusters at 400×magnification. The presence of a visible blood vessel lumen was not required for a section to be defined as positive. The mean value of the MVD in three to five examined hot spots per section was then calculated and assigned to one of 4 levels: 0~3 (−), 3~7 (+), 7~12 (++), >12 (+++). The MVD mean value was used to classify each group of samples as “high” (++, +++) or “low” (−, +).

### Cell culture, plasmids, generation of stable cell lines, and prokaryotic PSG9 expression

SW620, HCT-116, and HT-29 cells were obtained from the American Type Culture Collection (Manassas, VA, USA), while SW-480 and RKO cells were obtained from the Culture Collection of the Chinese Academy of Sciences (Shanghai, China). Human umbilical vein endothelial cells (HUVECs) were purchased from Sciencell Research Laboratories (Carlsbad, CA, USA) and were cultured in Endothelial Cell Medium (ECM). Full-length PSG9 DNA was cloned into the pcDNA3.1-myc-his (−) plasmid (Invitrogen, Carlsbad, CA, USA) to generate a PSG9 expression plasmid. The PSG9-shRNA and scrambled shRNA control plasmids were purchased from GeneChem (Shanghai, China). SMAD2, SMAD3, and SMAD4 plasmids were purchased from Vigene Biosciences (Jinan, Shandong, China).

Stable cell lines with PSG9 overexpression and control vectors of HT-29 (HT-29-pcPSG9 and HT-29-pcDNA3.1) and HCT-116 (HCT-116-pcPSG9 and HCT-116-pcDNA3.1) cells were generated by transfection and were selected and maintained with neomycin (G418, 50 μg/ml). Stable SW-480 cells with PSG9 downregulation (SW-480-shPSG9) and controls (SW-480-shCon) were generated by stable transfection with PSG9 shRNA (shPSG9) and control shRNA (shCon); these were selected and maintained with puromycin (1 μg/ml). *E. coli* Trans BL21 (DE3) cells were transformed with the pET-21α vector containing the PSG9 gene to obtain PSG9 protein.

### RNA extraction and real-time quantitative PCR

Total RNA was extracted using TRIzol and was reverse transcribed using a TransScipt II cDNA synthesis SuperMix kit (TransGen, Beijing, China) for standard real-time PCR (RT-PCR) analysis. Real-time PCR was performed using a SYBR green detection system in an ABI 7500 qPCR machine (Life Technologies, NY, USA); 18S was used as an internal reference gene. The gene-specific primers are summarized in Supplementary Table S5.

### Cell proliferation and Transwell migration assays

A CCK8 assay (Cell counting kit-8, KeyGENBioTECH, Nanjing, China) and a Click-iT®EdU (5′-ethynyl-2′-deoxyuridine) Flow Cytometry Assay Kit (Invitrogen, OR, USA) were used to determine cell proliferation abilities. For the colony formation assays, cells were seeded into 6-well plates and grown for 21 days. The migration assay was performed in Transwell chambers containing polycarbonate filters (BD Biosciences, CA, USA).

### HUVEC Matrigel tube-forming assay

Initially, Matrigel (BD Biosciences) was polymerized (200 μl/well of a 48-well culture plate) for 30 minutes at 37°C. Serum-starved HUVECs were resuspended in conditioned medium and reseeded in Matrigel-coated wells (10^5^ cells/well). The purified PSG9 protein, VEGFA and PSG9 antibody (5 μg/ml) were incorporated into the ECM and cultured with HUVECs. After 24 hours, images of capillary-like networks were recorded using a phase-contrast microscope. Images were analyzed with the Angiogenesis Analyzer plugin for Image J (Carpentier G, et al. Angiogenesis analyzer for ImageJ. 4^th^ ImageJ User and Developer Conference Proceedings), which is available on the NIH website (http://image.bio.methods.free.fr/ImageJ/?Angiogenesis-Analyzer-for-ImageJ&artpage=3-5).

### Xenograft model analysis

Animal experiments were performed in strict accordance with the recommendations in the Guide for the Care and Use of Laboratory Animals. The protocol was approved by the Committee on the Ethics of Animal Experiments of Beijing Chaoyang Hospital, Capital Medical University (Beijing China). Twenty male BALB/c nude mice (~6 weeks old) were purchased from Vital River (Beijing, China). Each mouse was inoculated (2×10^7^ cells per mouse) subcutaneously in the back adjacent to the left lower limb. Tumor dimensions were measured every 3-6 days. Mice were sacrificed at 38 days (~5 weeks) after injection, and the tumors were surgically isolated. IHC staining for CD31 in xenograft tumor tissues was performed according to the method described above.

### Western blotting

In all, 30 μg of cell lysates was subjected to sodium dodecyl sulfate polyacrylamide gel electrophoresis (SDS-PAGE) and transferred to polyvinylidene fluoride (PVDF) membranes (Millipore, MA, USA). The membranes were blocked in 5% milk and then incubated with various antibodies followed by incubation with the appropriate secondary antibodies. The antibodies used in the present study are summarized in Supplementary Table S6.

### Immunofluorescence and confocal fluorescence microscopy

Cells seeded on chamber slides were fixed in ice-cold formaldehyde, blocked with 5% bovine serum albumin and incubated with anti-PSG9 (1:100) and anti-SMAD4 antibodies (1:50) overnight at 4°C. Antibody-bound cells were detected by Cy3- or FITC-conjugated secondary antibody (Supplementary Table S6). DAPI was used to stain the nuclei, and the mounted slides were viewed under a Leica TCS SP5 confocal microscope (Leica Microsystems, Germany).

### Immunoprecipitation

Cell lysates were incubated with 2 μg of antibody and placed on a rotator overnight at 4°C. After extensive washing in RIPA lysis buffer, the immunoprecipitated complexes were resuspended in reducing buffer and boiled for 10 minutes. After centrifugation to pellet the Protein A/G Agarose beads (Santa Cruz), the supernatants were subjected to SDS-PAGE and immunoblotting.

### Chromatin immunoprecipitation

A chromatin immunoprecipitation (ChIP) assay kit was provided by Beyotime (Changsha, Hunan, China) and was used according to the manufacturer's instructions. The primers for ChIP were described in Supplementary Table S5.

### Angiogenic factor array

The expression of angiogenesis-related proteins in cell lysates was determined using a Human Angiogenesis Array Kit (R&D Systems Ltd., Abingdon, UK), which consists of 55 antibodies against angiogenesis-related proteins spotted onto a nitrocellulose membrane. The array was performed according to the manufacturer's instructions using 300 μg of cell lysates. The data from developed X-ray film were digitalized with a transmission-mode scanner and were quantified using Image J analysis software as described previously [39]. The average signal background was subtracted, and the arrays were calibrated based on the signal strength of the positive reference. The average signal (pixel density) of the pair of duplicated spots representing each angiogenesis protein was determined, and the corresponding signals on different arrays were compared.

### Statistics

The significance of the data from patient specimens was determined by the χ^2^ test or Mann-Whitney *U* test. The significance of the *in vitro* and *in vivo* data was determined by Student's *t* test. Overall survival (OS) rates were assessed by the Kaplan-Meier test, and the log-rank test was used to plot survival curves. *P*<0.05 was considered to be significant.

## SUPPLEMENTARY FIGURES AND TABLES


