# Supplementary material for: Pregnancy-specific glycoprotein 9 (PSG9), a driver for colorectal cancer, enhances angiogenesis via activation of SMAD4
Source: Oncotarget. 2016 Aug 9;7(38):61562–74. doi: 10.18632/oncotarget.11146 (PMC5308672; doi:10.18632/oncotarget.11146)
Supplement: Supplementary file 1 [file oncotarget-07-61562-s001.pdf]

Pregnancy-specific glycoprotein 9 (PSG9), a driver for colorectal cancer, enhances angiogenesis via activation of SMAD4

SUPPLEMENTARY FIGURES AND TABLES

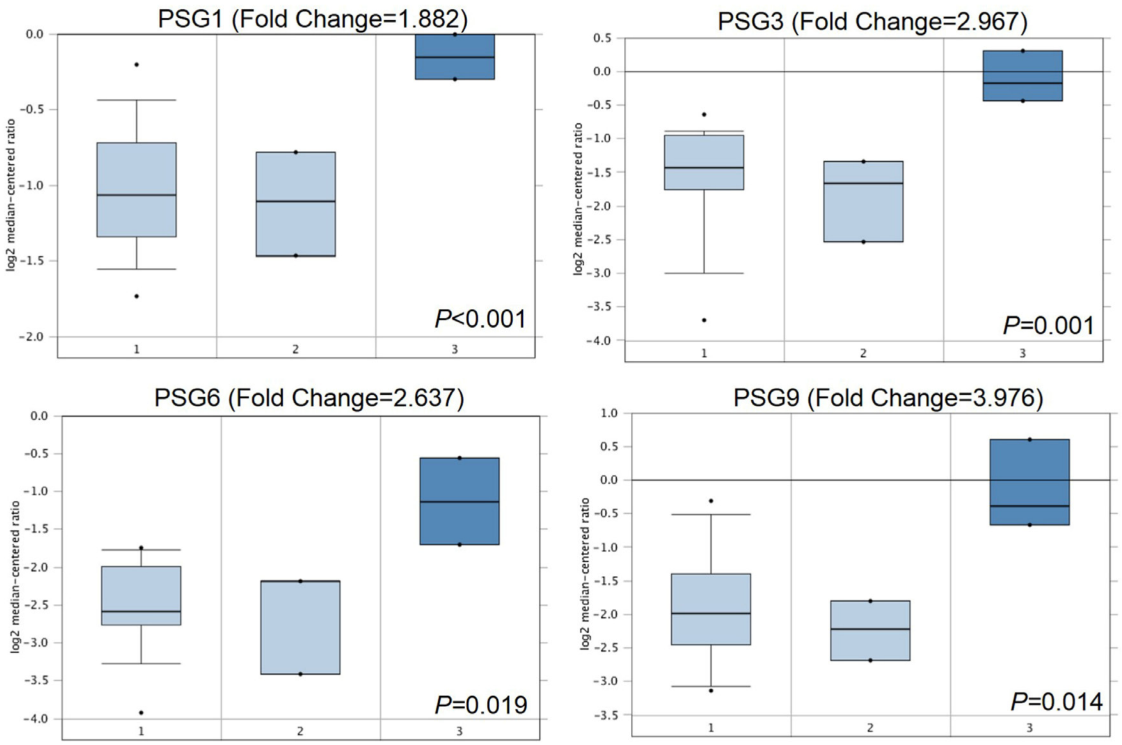

Supplementary Figure S1: The Oncomine microarray database (https://www.oncomine.org) was searched to analyze the mRNA expression of PSG1, PSG3, PSG6, or PSG9 in patients with CRC. 1, Colon tissues, n=19; 2, Rectal tissues, n=3; 3, Rectosigmoid adenocarcinoma, n=3.

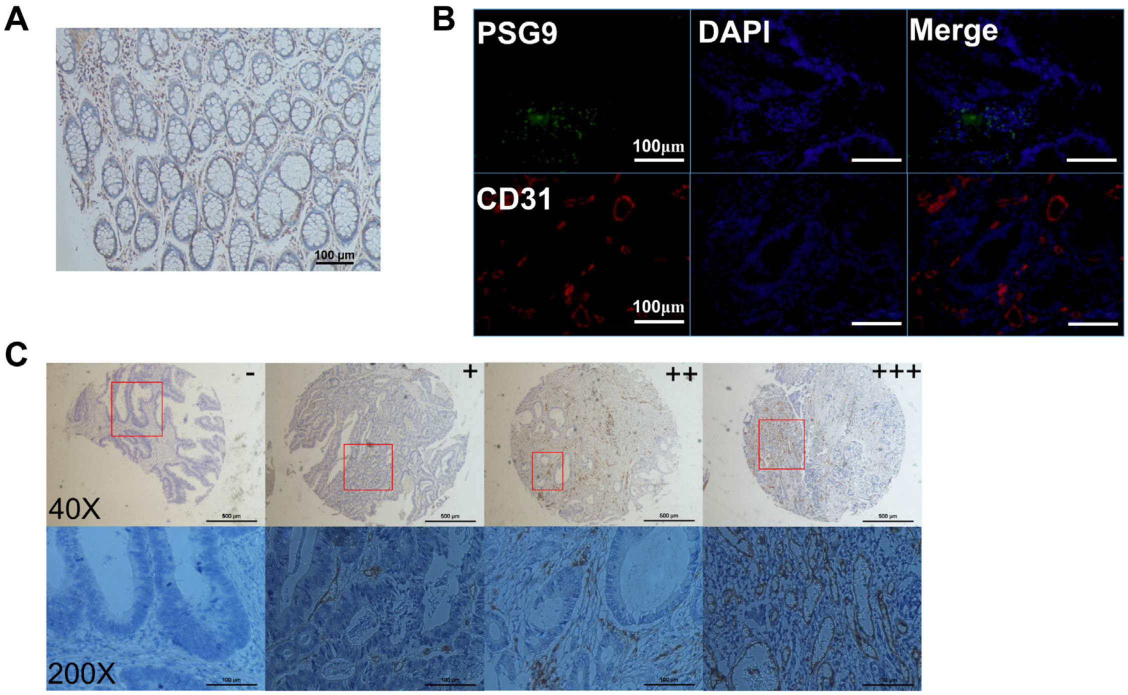

Supplementary Figure S2: PSG9 are associated with micro-vessel density (MVD). A. Immunohistochemical staining shows a relative low PSG9 expression in a paired normal CRC tissue section. **B.** Immunofluorescence (IF) staining for PSG9 and CD31 in CRC tissues. **C.** Representative images of negative (-), weak (+), moderate (++), and strong (+++) staining for CD31 in CRC tissues.

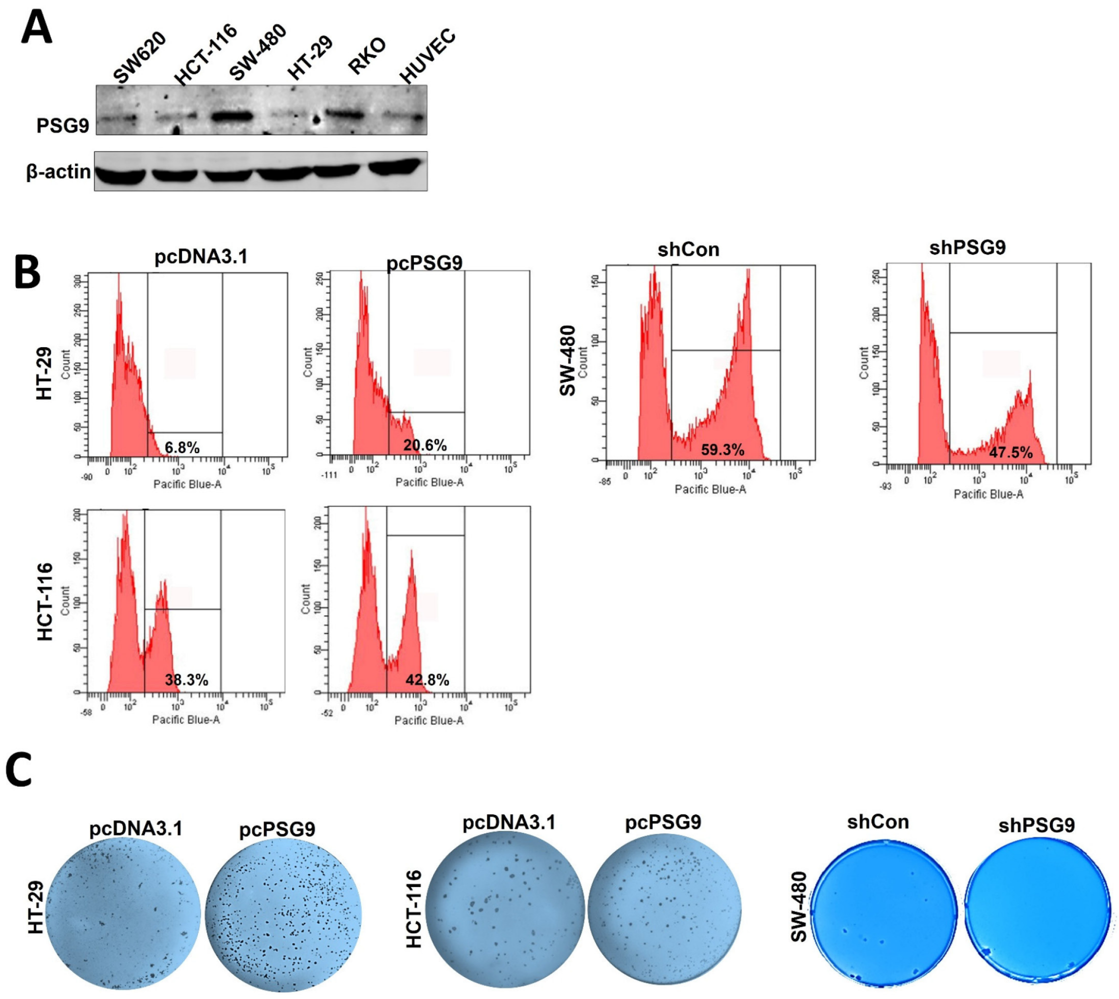

Supplementary Figure S3: PSG9 promotes the proliferation of CRC cells. A. Western blotting identified the expression of PSG9 in SW620, HCT-116, SW-480, HT-29, RKO, and HUVEC cells. **B.** Representative flow cytometry (FCM) images used to determine the proliferating cells by EdU assay. **C.** Colony formation assays determined the effect of PSG9 expression on the colony-forming ability of the cells.

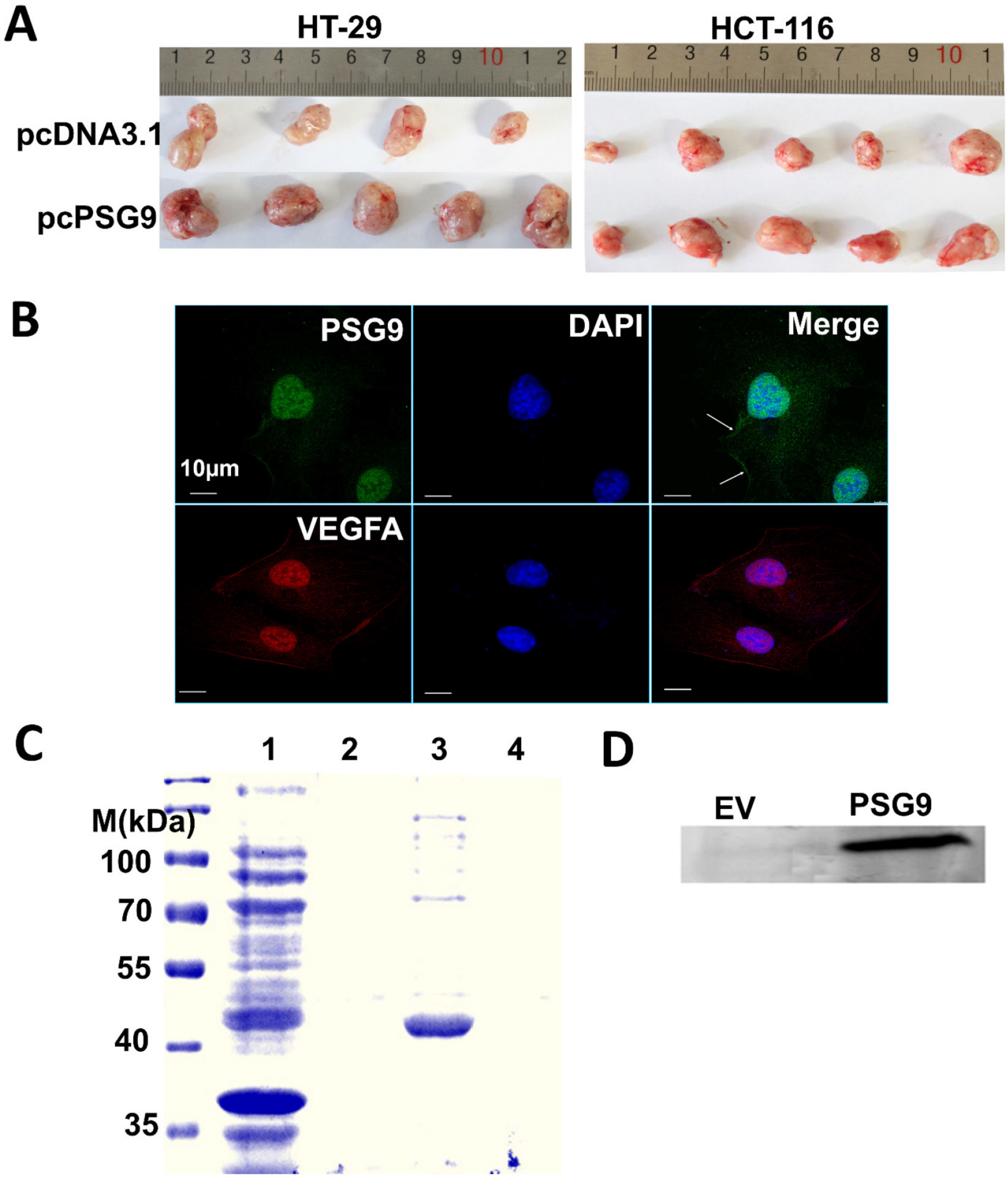

Supplementary Figure S4: Tumor xenograft models of HT-29-pcDNA3.1, HT-29-pcPSG9, HCT-116-pcDNA3.1, and HCT-116-pcPSG9 cells and the prokaryotic expression of PSG9. A. The tumors that formed in the mice. **B.** Immunofluorescence (IF) staining for PSG9 and VEGFA in HUVECs. **C.** Purified PSG9, elution by a concentration gradient of imidazole (1, pre-purified *E.coli* lysates; 2, 20 nM, 3, 40 nM, 4, 100 nM). **D.** Western blotting identified purified PSG9 proteins. EV, emptor vector.

Supplementary Table S1: Serum PSG9 levels and basic clinical characteristics

	PSG9 levels (mean ± SD) (μg/ml)	*P* value
**Healthy controls (n=125)**	0.90±1.92	
Sex		
Male (n=56)	0.78±1.43	
Female (n=69)	0.99±2.25	0.320
Age, years		
<55 (n=86)	1.00±2.19	
≥55 (n=39)	0.68±1.13	0.210
**CRC(n=140)**	3.40±10.59	
Sex		
Male (n=72)	2.02±3.56	
Female (n=68)	4.86±14.66	0.830
Age, years		
<55 (n=42)	1.96±6.56	
≥55 (n=98)	4.03±11.88	0.060
Tumor size		
≤5 cm (n=31)	1.11±1.77	
>5 cm (n=88)	4.81 ± 13.13	0.037
Missing (n=21)		
Differentiation		
Well (n=3)	0.68±0.38	
Moderate (n=86)	4.11±12.41	
Poor (n=31)	2.75±8.63	0.627
Missing (n=20)		
T stage		
T1-2 (n=31)	2.66±8.28	
T3-4 (n=102)	2.86±10.69	0.499
Missing (n=7)		
N stage		
N0 (n=81)	1.89±3.38	
N1 (n=47)	6.55±17.40	
N2 (n=9)	1.29±2.44	0.591
Missing (n=3)		

SD, standard deviation

Supplementary Table S2: Association of clinical parameters and PSG9 levels in CRC tissues

	PSG9 staining				χ2	*P* value
	-	+	++	+++		
Age(years)						
≤66 (n=37)	1 (2.70%)	8 (21.62%)	13 (35.14%)	15 (40.54%)	0.76	0.782
>66 (n=37)	1 (2.70%)	7 (18.92%)	13 (35.13%)	16 (43.24%)		
Sex						
Female (n=34)	0 (0%)	6 (17.65%)	15 (44.12%)	13 (38.24%)	1.01	0.315
Male (n=40)	2 (5.00%)	9 (22.50%)	11 (27.50%)	18 (45.00%)		
TNM						
I-II (n=50)	1 (2.00%)	11 (22.00%)	18 (36.00%)	20 (45.83%)	0.09	0.762
III-IV (n=24)	1 (4.17%)	4 (16.67%)	8 (33.33%)	11 (45.83)		
Tumor size*						
≤5 cm (n=32)	1 (3.13%)	7 (21.88%)	13 (40.63%)	11 (34.38%)	0.13	0.718
>5 cm (n=41)	1 (2.44%)	8 (19.51%)	12 (29.27%)	20 (48.78%)		
Lymph node						
Negative (n=52)	1 (1.92%)	11 (21.15%)	18 (34.62%)	22 (42.30%)	0.00	0.974
Positive (n=22)	1 (4.55%)	4 (18.18%)	8 (36.36%)	9 (40.91%)		
Differentiation						
Well (n=21)	2 (9.52%)	3 (14.28%)	7 (33.33%)	9 (42.86%)	0.01	0.914
Moderate/Poor (n=53)	0 (0%)	12 (22.64%)	19 (35.85%)	22 (41.51%)		

*one subject missing

Supplementary Table S3: Micro-vessels density (MVD) in CRC patients with low or high PSG9 levels

PSG9 levels	MVD				χ^2	*P* value
	-	+	++	+++		
Low (n=43)	14 (32.56%)	12 (27.91%)	7 (16.27%)	10 (23.26%)	4.50	0.034
High (n=31)	6 (19.35%)	5 (16.13%)	14 (45.16%)	6 (19.36%)		

Supplementary Table S4: Cox regression analysis factors associated with overall survival

Variates	Univariate		Multivariate	
	HR (95% CI)	*P*	HR (95% CI)	*P*
Tissue PSG9<br>Low vs. High	2.803<br>(1.238-6.349)	0.013	2.033<br>(0.856-4.830)	0.108
MVD<br>Low vs. High	2.382<br>(1.027-5.525)	0.043	1.609<br>(0.631-4.100)	0.319
Age(years)<br>≤66 vs. >66	0.901<br>(0.411-1.974)	0.794		
Sex<br>Female vs. Male	1.967<br>(0.848-4.562)	0.115		
TNM<br>I-II vs. III-IV	4.208<br>(1.879-8.423)	<0.001	6.789<br>(1.292-35.68)	0.024
Tumor size<br>≤5cm vs. >5cm	2.955<br>(1.178-7.416)	0.021	1.588<br>(0.549-4.422)	0.405
Lymh node<br>N vs. P*	3.234<br>(1.470-7.114)	0.004	0.524<br>(0.109-2.524)	0.524
Differentiation<br>W vs. M/P#	1.766<br>(0.663-4.707)	0.255		

MVD, Microvessel Density; CI, confidence interval; HR, hazard ratio; * N, negative; P, Positive; #, W, well; M, Moderate; P, Poor

Supplementary Table S5: Primers used in this study

Genes		Sequence
		**Primers for real time PCR**
PSG9	Up	GCGAGGTGATGAGACTAGAGA
	Down	GGTTTTGGACAGCTGCAACC
VEGFA	Up	5’-TGCTTCTGAGTGCCCAGGA-3’
	Down	5’-TGGTTCAATGGTGTGAGGACATAG-3’
IGFBP-3	Up	5’-CGCCAGGAAATGCTAGTGAG-3’
	Down	5’-ATGTGTACACCCCTGGGACT-3’
PDGF-AA	Up	5’-TCGATGAGATGGAGGGTCG-3’
	Down	5’-ACCCGGACAGAAATCCAGTCT-3’
GM-CSF	Up	5’-AATGTTTGACCTCCAGGAGCC-3’
	Down	5’-GGTGATAGTCTGGGTTGCACA-3’
18S	Up	5‘-AAA CGG CTA CCA CAT CCA-3’
	Down	5‘-CAC CAG ACT TGC CCC TCC A-3’
		**Primers for ChIP**
VEGFA	Up	5‘-ACCTAGCACCTCCACCAAAC-3’
	Down	5‘-AAACGCTCCAGGGAGCTTAC-3’
IGFBP-3	Up	5‘-TGTATGCCAGTTTCCCCGAC-3’
	Down	5‘-CCGGGTCACCTTGTCGTCTA-3’
PDGF-AA	Up	5‘-GAAATGTGGGTGGAGGGTCC-3’
	Down	5‘-CCCAGCCTTCCTCTCTCCAT-3’
GM-CSF	Up	5‘-TGAGATGGATGCAGCCACAG-3’
	Down	5‘-GAGCCACCTTCCTGAGTGAC-3’

Supplementary Table S6: Antibodies used in this study

Antibody	Dilution	Clone, source	Company
PSG9	WB1:1000	Rabbit polyclonal	Abcam, Cambridge, MA, USA
SMAD2/3	WB1:200	Mouse monoclonal	Santa Cruz, CA, USA
SMAD4	WB1:500	Mouse monoclonal	Santa Cruz
GAPDH	WB1:3000	Mouse monoclonal	Beyotime Biotechnology, Changsha, Hunan, China
β-actin	WB1:2000	Mouse monoclonal	Beyotime Biotechnology
LaminB1	WB1:100	Rabbit polyclonal	Cell Signaling Technologies, Danvers, MA, USA)
CD31	IHC 1:200	Rabbit monoclonal	Zhongshan Golden Bridge, Beijing, China
Anti-c-Myc-tag	WB 1:1000	Rabbit polyclonal	CWBIO, Beijing, China
Anti-Flag-tag	WB 1:500	Mouse monoclonal	CWBIO
IRDye 680RD anti-mouse IgG (H+L)	WB 1:10000	Goat	LI-COR, NE, USA
IRDye 680RD anti-rabbit IgG (H+L)	WB 1:10000	Goat	LI-COR
FITC-conjugated goat-anti-rabbit secondary antibody	IF 1:100	Goat	CUSABIO, Wuhan, China
Cy3-conjuated goat-anti-mouse secondary antibody	IF 1:100	Goat	CUSABIO

Immunofluorescence, IF; Western blotting, WB; Immunohistochemistry, IHC.
